# Automating Individualized Notification of Drug Recalls to Patients: Complex Challenges and Qualitative Evaluation

**DOI:** 10.2196/68345

**Published:** 2026-01-13

**Authors:** Meghana Gadgil, Rose Pavlakos, Simona Carini, Brian Turner, Ileana Elder, William Hess, Lisa Houle, Lavonia Huff, Elaine Johanson, Carole Ramos-Izquierdo, Daphne Liang, Pamela Ogonowski, Joshua Phipps, Tyler Peryea, Ida Sim

**Affiliations:** 1 Division of General Internal Medicine, University of California, San Francisco, Box 0320, San Francisco, CA, 94143, United States, 1-415-353-7922; 2 Division of Cardiology, University of California, San Francisco, San Francisco, CA, United States; 3 Clinical and Translational Science Institute, University of California, San Francisco, San Francisco, CA, United States; 4 Food and Drug Administration, Silver Spring, MD, United States; 5 Tuvli, LLC, Herndon, VA, United States; 6 Conceptant, Inc, Falls Church, VA, United States

**Keywords:** notification system, drug recalls, patient safety, medication, electronic health records, prescriptions, decision support

## Abstract

**Background:**

Consumer-level drug recalls usually require action by individual patients. The Food and Drug Administration (FDA) has public-facing outlets to inform the public about drug safety information, including all recalls, but individual consumers may not be aware of them. And there is no system in place to notify individual prescribers which of their patients are affected by a specific recall.

**Objective:**

We aimed to leverage the FDA’s Healthy Citizen prototype web-based software platform, which provides users with information about recalls, to automatically notify patients of relevant recalls.

**Methods:**

We developed and evaluated an electronic notification system in the primary care and cardiology practices at a large, urban, academic medical center. The health care portal scanned the FDA Healthy Citizen application programming interface nightly to detect new recalls, identified patients who had those medications in their electronic health record (EHR) medication list, and sent them a message through the EHR patient portal with a link to a customized FDA information display. Using structured interviews, we assessed qualitative feedback on the system and portal messaging from a convenience sample of 9 patients.

**Results:**

The system was technically functional, but it was not possible to trace a medication prescription from the EHR to specific lot numbers dispensed to that patient by a community pharmacy. The qualitative feedback obtained from patients showed convergence of topics.

**Conclusions:**

Lack of an accurate electronic audit trail from prescription to dispensed medication precludes clinical deployment of automated drug recall notification.

## Introduction

### Background

In the United States, the Food and Drug Administration (FDA) is responsible for assuring the safety and efficacy of marketed drugs. When a safety concern arises on a marketed drug, communicating this information to patients is essential, and timely clinical action by prescribers is often required. Yet, patients and prescribers often lack relevant, timely information, leaving patients and health systems unable to efficiently manage drug recalls and their impacts. Recognizing this problem, the FDA developed prototype technology for patients and health systems to automatically be notified of drug recalls through their health care portals as part of the FDA’s Healthy Citizen prototype platform that seeks to allow “citizens and those who care for them, research organizations, and FDA to communicate and collaborate in a single, seamless environment connected through the healthcare portal and leveraging the trusted relationships between providers and patients to improve public health outcomes” [1].

### Drug Recall Process

Firms, including manufacturers and own-label distributors, can initiate a recall, either on their own or in response to an FDA recommendation, request, or order. Common reasons for recalls include contamination, mislabeling, adverse reactions, defective products, and incorrect potency [2,3]. The FDA works with firms as they develop their recall strategy, which is dependent on a variety of factors, including, but not limited to, the product’s degree of hazard, the ease of identifying the product, and the extent of distribution. Depth of recall is one component of this strategy: consumer-level recalls should be extended to consumers and patients; retail-level recalls affect community pharmacies and health care providers; and wholesale-level recalls affect manufacturers and distributors.

For consumer-level recalls, which were the focus of this project, consumers may learn of a recall through FDA.gov [4], news media, or notification from the recalling firm or pharmacy. (Most pharmacies have protocols in place to handle recalls, which may include outreach to customers.) Consumer notifications often recommend that patients consult their health care provider about the best course of action. However, recalls often affect only certain lots of pills, and prescribers have no way of knowing the lot number of the medication dispensed to the patient and therefore whether the patient is affected. The patient often cannot identify the lot number, either, as most dispensing pharmacies are not required to document the lot number on pill bottle labels (see Principal Findings section for details). Thus, if patients contact their health care providers about a recall, the only action providers can take is to redirect patients to their pharmacy. The pharmacy then either replaces the pills with those from an unaffected lot or, if no substitute is available, notifies the prescribing clinician to issue a new prescription for a different medication, dosage, or formulation.

This partnership with the FDA aimed to address the inefficiencies in recall notification by demonstrating timely, fully automated, and individualized communication of drug recalls and recommended actions to patients.

## Methods

### Study Setting and Participants

The University of California, San Francisco (UCSF), an academic medical center, partnered with the FDA [5] to demonstrate use of Healthy Citizen tools to automate individualized drug recall notifications to outpatient primary care and cardiology patients.

We developed an electronic notification system and conducted this study in the Division of General Internal Medicine (DGIM) and Division of Cardiology at UCSF, a large, urban, academic medical center in San Francisco, California. The DGIM primary care clinic serves 25,000 patients with approximately 70,000 visits yearly. The cardiology clinics serve over 12,000 patients with approximately 30,000 visits yearly. The clinics use the Epic electronic health record (EHR) with the MyChart patient portal. At the time of the study, approximately 45% of patients had actively used MyChart at least once.

We created and tested the notification system within Epic’s ACE6 development environment, with the intent to migrate it to production after successful testing. The project team comprised clinicians and programmers from the medical center, FDA leaders from the Office of Health Informatics and other sections, and developers of the Healthy Citizen platform. For testing purposes, fictitious patients were created in the Epic ACE6 environment with medication lists that contained prescriptions matching fictitious medication recalls issued by the FDA.

The prototype system was shown to a convenience sample of 9 patients via remote videoconferencing to obtain initial formative feedback.

### Ethical Considerations

Ethical approval for this program evaluation was obtained from the UCSF Institutional Review Board (19‐27668). Informed consent was obtained from each participant via electronic signature before the interview. Interviews were conducted by 2 investigators (MG and RP). Transcripts were analyzed for common themes by the same, with additional verification by 2 other investigators (SC and IS). Transcripts were stored in a secure location behind an institutional firewall. No identifying data were shared or presented beyond summary statistics (number and gender of patients). Upon completion of the interview, each patient was paid US $25 for their time.

## Results

### Program Description

The notification system comprised two major technical parts. The first part, within the medical center’s firewalls, checked for new consumer-level drug recalls and notified affected patients via MyChart (see the EHR Build section below). The second part was the FDA’s Healthy Citizen prototype platform, which provided an application programming interface (API) for external systems to request the latest drug recall information and mechanisms to launch a widget displaying details about a specific recall (see the Healthy Citizen Build section below). The widget was a SMART-on-FHIR software module that could be embedded into and accessed within an EHR without the need for any additional sign-in.

### EHR Build

The EHR build had three major parts: (1) checking for new drug recalls; (2) matching recalls to the patient medication lists; and (3) preparing and sending personalized MyChart notifications to patients. Each part proved extremely challenging to build for technical and data availability reasons.

First, the system issued a nightly call to the Healthy Citizen API to retrieve the National Drug Codes (NDCs) of newly recalled drugs. The next step, matching recalls to a patient’s EHR medication list, can result in false negatives and false positives. False negatives can occur if a patient’s prescription is missing from the medication list [6], or if the algorithm fails to detect a true match. False positives can arise from two inaccuracies. Crucially, EHR medication lists contain the *prescribed* drug, not the *dispensed* drug. To identify a prescribed drug, Epic uses RxNorm codes that do not include the manufacturer name. To identify recalled drugs and their manufacturers, the FDA uses NDCs, which are unique, 3-segment numbers that identify a drug’s labeler (ie, manufacturer or distributor), product, and trade package size [7]. Thus, for example, the NDC from a lisinopril recall from a specific manufacturer will match the RxNorm code for all lisinopril prescriptions of the same strength, regardless of manufacturer. This will erroneously identify patients who were prescribed lisinopril but were not dispensed pills from the affected manufacturer. Secondly, recalls often involve only specific lots, information that is unavailable in the EHR, thereby contributing to false positives, as discussed above.

**Figure 1.**
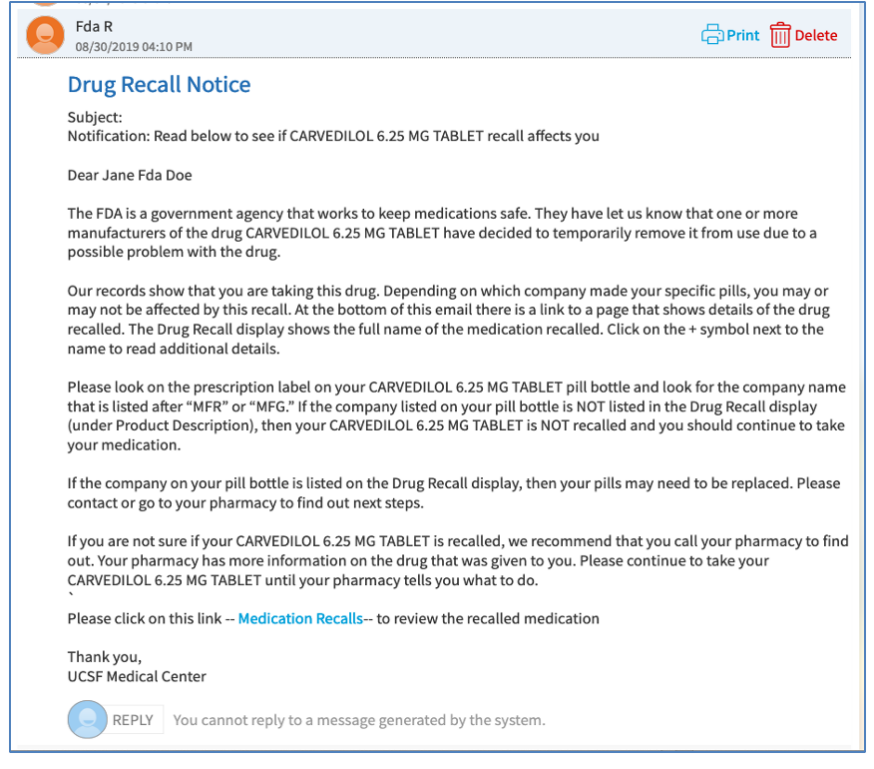
MyChart notification of potentially relevant recall.

The third part of the EHR build was to send a MyChart notification to patients once a match was made, alerting them that they may be taking a recalled medication (Figure 1).

**Figure 2.**
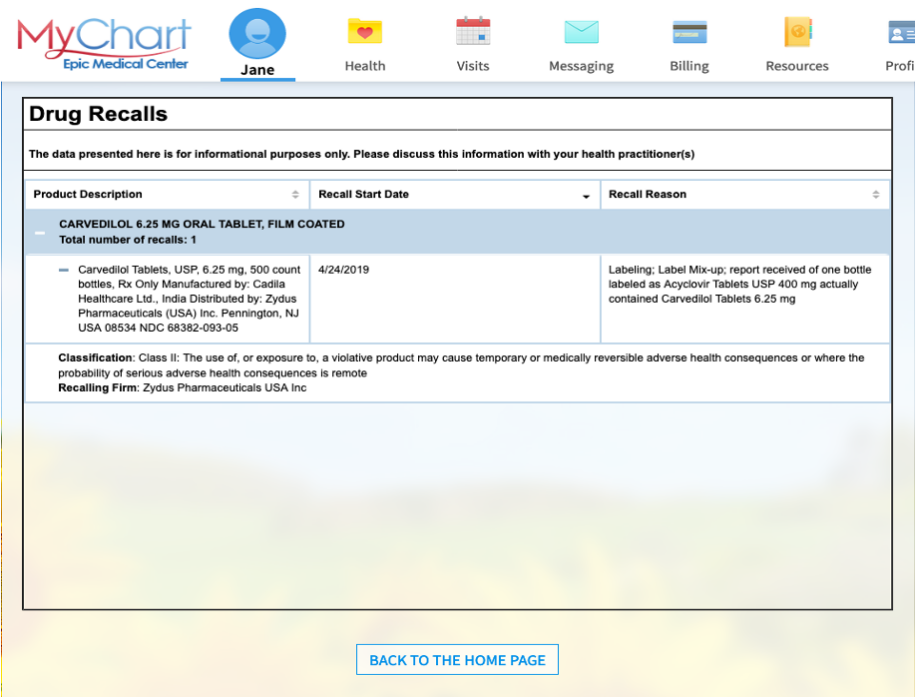
Food and Drug Administration Healthy Citizen information display widget showing official information about a drug recall.

The Medication Recalls link led to the FDA Healthy Citizen’s display widget, launched as a new window within MyChart showing details of the matched recall, including affected manufacturers (Figure 2). Because the matching algorithm could not restrict matches to affected manufacturers, the MyChart message asked patients to compare the manufacturer name on their pill bottle’s label to the manufacturer or manufacturers listed in the FDA informational display and to call their pharmacy if it matched. The patient advisory council of the primary care clinic provided input on the wording and endorsed the importance of the project aims.

### Healthy Citizen Build

Substantial technical work was performed on the Healthy Citizen platform to satisfy the use case needs. For example, technical and internal FDA administrative changes were required to make the depth of recall (ie, retail or consumer level) searchable and to distinguish between new and ongoing recalls. The SMART-on-FHIR widget needed to be available on Epic’s App Orchard, and modifications were required for the widget to be called by and launched within Epic. The contents of the widget display were modified to exclude information not relevant to patients, such as the status of the recall (eg, whether it was ongoing or completed), or to move it from the main display to the Additional Details section. The text immediately below the title could not be modified.

### Program Evaluation

The system was able to automatically detect a new fictitious medication recall using the Healthy Citizen API, compare and detect matches to each (fictitious) patient’s list of prescribed medications, send a MyChart message to affected (fictitious) patients, and launch a display for the correct recall or recalls. The system responded correctly to test patients with zero to multiple affected medications.

Established patients at the primary care clinic who were members of the patient advisory council, used MyChart, and were prescribed at least one medication received a recruitment letter. Patients at the cardiology clinics who were scheduled to see the pharmacist during a random week, who actively used MyChart (or their family members who used MyChart on their behalf), and who were taking at least one prescription medication were deemed eligible for the study and sent a recruitment letter. Interested patients contacted the study team to participate.

We obtained qualitative feedback by interviewing a convenience sample of 9 patients (5 female, 4 male). Two of the 9 participants had personal experience with recalls. Participants were interviewed individually using Zoom (Zoom Communications, Inc). During the session, they were presented with a scenario for fictitious patient Jane Doe, who was prescribed carvedilol (6.25 mg). Using structured interviewing techniques, we evaluated participants’ understanding of the MyChart message, widget, and example pill bottle label. Throughout the process we asked for descriptive feedback. Recordings of the interviews were transcribed and separately analyzed by 2 investigators (MG and RP) for common themes [8], with additional verification by SC and IS.

All 9 participants understood the purpose of the MyChart notification message but thought it was too wordy. All 9 were able to identify the medication manufacturer on the example pill bottle label. Only 2 would have clicked on their own on the link at the bottom of the MyChart message to launch the widget; the other 7 needed the interviewer’s prompting and guidance to do so. As advised by the MyChart message, all 9 users would have contacted their pharmacist, but 5 of the 9 would also have contacted their doctor’s office, as advised by the widget. Given the choice, all 9 would have liked to receive MyChart notification of potential drug recalls.

Major thematic findings included the following: (1) Patients appreciated being notified of recalls by their clinic, even though their actual medication may not have been affected by the recall, because they trusted the clinic, and the notification showed that the clinic was aware of patient medication issues. (2) Patients saw communicating through the MyChart patient portal as a trusted, efficient, and reliable notification method. Mailed letters can be ignored, and several users said they did not answer phone calls from unknown phone numbers (eg, their pharmacy). (3) Patients suggested that the widget content should be displayed directly in the MyChart message rather than in a new window. (4) Patients felt that the widget itself should be redesigned to more directly meet patient information needs (much of the widget content was either confusing or irrelevant to patients, eg, recall start date, manufacturer address), that the recall reason was appreciated but unnecessary, and that the widget should not ask patients to discuss the information with their health care provider. (5) Patients wanted to discuss the recall with their clinicians to “close the loop.”

The project team concluded that operational deployment of this system may lead to unnecessary and unacceptable patient anxiety generated by false positive notifications. In addition, because patient feedback suggested that patients would contact their clinicians regardless of the advice to contact their pharmacy, the system was likely to increase staff burden for responding to patient inquiries. While the project implementation provided important lessons, it did not provide a solid enough business case to justify expanding the pilot, which would have required institutional support. We therefore decided not to proceed with implementation of the FDA drug recall notification system into clinical care.

## Discussion

### Principal Findings

Drug recalls are an ongoing challenge in the United States [3] and other countries [9]. According to an analysis of FDA recall data, between 2012 and 2023 there were on average 330 recalls per year [3]. When, in 2018, several angiotensin II receptor blockers (prescribed to treat hypertension, heart failure, and chronic kidney disease) were recalled for carcinogenic impurities, the availability of treatments in the same or similar drug class facilitated patients’ transition to alternatives [10]. Sustained media attention highlighted communication needs and challenges among the parties impacted.

Patients and clinicians need an accurate system for identifying which patients are affected by which drug recalls and acting on them in a timely and appropriate manner to prevent patient harm and erosion of trust in prescribers and the health care system.

This project demonstrated the technical and clinical feasibility of using the FDA’s Healthy Citizen drug recall tools to automatically alert patients, via Epic’s patient portal MyChart, to relevant drug recalls. While our project was technically successful, it revealed substantial challenges to responding to drug recalls. Chiefly, while patients want and expect their prescriber to be aware of, and involved in, responding to a drug recall, prescribers have no easy access to the manufacturer and lot number of the actual medication dispensed to their patients. Without these details, health systems cannot accurately target patients and false positive notifications are inevitable. A partial technical solution could be to access Surescripts records, which include the NDC for dispensed drugs as reported to Surescripts via claims data. However, only 70% of patients at UCSF Medical Center use a Surescripts-participating pharmacy, and Surescripts records do not include dispensed lot numbers, such that false positive recall notifications would still be an issue.

Our project showed that a strong case can be made for requiring each pill bottle to include on its label the lot number and NDC of the pills (which links to the manufacturer, labeler, or distributor), so that patients could definitively determine if a recall affected them. Current federal regulation allows such information to appear on an internal leaflet or a label on the outer carton or wrapper of manufactured medications [11], which many patients discard even if the pharmacist includes them with the dispensed medication. As of August 2025, our review of state regulations identified jurisdictions with explicit requirements. Only four state boards of pharmacy (Colorado, Delaware, Oklahoma, and Wyoming), plus the US territory of Puerto Rico, require the lot number to appear on the dispensed medication label [12-16]. In addition, only three state boards of pharmacy (Pennsylvania, New Hampshire, and Ohio) have regulations about the NDC appearing on the dispensed medication label [17-19]. The Pennsylvania State Board of Medicine requires the NDC to appear on the dispensed medication label if the prescriber specifies that the drug name *not* appear on the label [17]. The state boards of pharmacy of New Hampshire and Ohio allow the use of the NDC as an abbreviation for the manufacturer or distributor name, though they do not require it on every dispensed medication label [18,19]. The FDA does not have the legal authority to regulate the practice of pharmacy in any state and therefore cannot require that the lot number and NDC (or anything else, including the name of the drug) be placed on each prescription that a pharmacist dispenses to a patient. The manufacturer and lot number of dispensed medications should routinely be available electronically to prescribing clinicians via standard APIs so that health systems can meet patient expectations that they are trusted guides in properly responding to drug recalls. Policy and data infrastructure changes are required at the regulatory, health IT, and consumer pharmacy levels before automated recall notification can be widely deployed.

### Conclusions

The need of patients and clinicians to identify applicable drug recalls and appropriately act on them is currently unmet. Through our project we learned several lessons, which in some cases can be generalized beyond its scope: (1) Patients appreciated receiving a notification showing that the clinic was aware of the patient’s medication issues. (2) The MyChart patient portal was seen as a trusted and reliable notification method. (3) Patients preferred the notification content to be displayed directly in the MyChart message rather than in a new window. (4) Patients considered that the content of the notification should directly address patient information needs, avoiding content that is not strictly necessary. (5) Prescriptions being a sensitive topic, patients wished to discuss the recall with their clinicians, even when directed to contact the dispensing pharmacy.

Our project showed that access to the manufacturer and lot number of the drug dispensed via standard APIs is a requirement for the development and deployment of technical solutions that implement accurate automated recall notifications to patients. While a change at the federal level would be ideal, advocating for individual state boards of pharmacy to require the NDC and lot number to appear on the dispensed medication label may provide needed interim progress for allowing development and deployment of solutions supporting patients’ needs.

## Abbreviations

API: application programming interface
DGIM: Division of General Internal Medicine
EHR: electronic health record
FDA: Food and Drug Administration
NDC: National Drug Code
UCSF: University of California, San Francisco
